# Molecular Targets of the 5-Amido-Carboxamide Bumped Kinase Inhibitor BKI-1748 in *Cryptosporidium parvum* and HCT-8 Host Cells

**DOI:** 10.3390/ijms25052707

**Published:** 2024-02-26

**Authors:** Jubilee Ajiboye, Anne-Christine Uldry, Manfred Heller, Arunasalam Naguleswaran, Erkang Fan, Wesley C. Van Voorhis, Andrew Hemphill, Joachim Müller

**Affiliations:** 1 Institute of Parasitology, Vetsuisse Faculty, University of Bern, Länggass-Strasse 122, 3012 Bern, Switzerland; jubilee.ajiboye@med.uvm.edu; 2 Cellular, Molecular and Biomedical Sciences Graduate Program, University of Vermont, Burlington, VT 05405, USA; 3 Proteomics and Mass Spectrometry Core Facility, Department for BioMedical Research (DBMR), University of Bern, Länggass-Strasse 122, 3012 Bern, Switzerland; anne-christine.uldry@dbmr.unibe.ch (A.-C.U.); manfred.heller@dbmr.unibe.ch (M.H.); 4 Institute of Molecular Pathology, Vetsuisse Faculty, University of Bern, Länggass-Strasse 122, 3012 Bern, Switzerland; arunasalam.naguleswaran@unibe.ch; 5 Department of Biochemistry, University of Washington, Seattle, WA 98109, USA; erkang@uw.edu; 6 Center for Emerging and Re-Emerging Infectious Diseases (CERID), Division of Allergy and Infectious Diseases, Department of Medicine, University of Washington, Seattle, WA 98109, USA; wesley@uw.edu

**Keywords:** affinity chromatography, binding proteins, proteomics, side effects, splicing

## Abstract

*Cryptosporidium parvum* is an apicomplexan parasite causing persistent diarrhea in humans and animals. Issuing from target-based drug development, calcium-dependent protein kinase 1 inhibitors, collectively named bumped kinase inhibitors (BKIs), with excellent efficacies in vitro and in vivo have been generated. Some BKIs including BKI-1748 share a core structure with similarities to the first-generation antiprotozoal drug quinine, which is known to exert notorious side effects. Unlike quinine, BKI-1748 rapidly interfered with *C. parvum* proliferation in the human colon tumor (HCT) cell line HCT-8 cells and caused dramatic effects on the parasite ultrastructure. To identify putative BKI targets in *C. parvum* and in host cells, we performed differential affinity chromatography with cell-free extracts from non-infected and infected HCT-8 cells using BKI-1748 and quinine epoxy-activated sepharose columns followed by mass spectrometry. *C. parvum* proteins of interest were identified in eluates from columns coupled to BKI-1748, or in eluates from both BKI-1748 and quinine columns. However, no *C. parvum* proteins could be identified binding exclusively to BKI-1748. In contrast, 25 BKI-1748-specific binding proteins originating from HCT-8 cells were detected. Moreover, 29 *C. parvum* and 224 host cell proteins were identified in both BKI-1748 as well as in quinine eluates. In both *C. parvum* and host cells, the largest subset of binding proteins was involved in RNA binding and modification, with a focus on ribosomal proteins and proteins involved in RNA splicing. These findings extend previous results, showing that BKI-1748 interacts with putative targets involved in common, essential pathways such as translation and RNA processing.

## 1. Introduction

Apicomplexan parasites, in particular *Plasmodium*, *Cryptosporidium*, *Eimeria*, *Babesia*, *Theileria*, *Sarcocystis, Toxoplasma, Besnoitia* and *Neospora*, can cause serious diseases in animals and humans. *Cryptosporidium* ssp., notably *C. hominis* and *C. parvum*, are the causative agents of persistent diarrhea, presenting serious health risks for immunocompromised persons and for children, particularly in combination with malnutrition [1,2]. Moreover, there is some evidence that cryptosporidiosis may be correlated to the formation of cancers of the digestive tract [3]. The transmission occurs via food or water contaminated with oocysts. Activated during transit through the stomach and small intestine, these oocysts liberate sporozoites, which infect intestinal cells and develop to merozoites. These merozoites proliferate asexually and re-infect other intestinal cells then undergo sexual development forming oocysts which are excreted via feces [1,4]. Thus, this obligate parasite completes its life cycle in a single host. Unique among apicomplexans and relevant to drug development, *C. parvum* lives in an intracellular (within host plasma membrane) but extra-cytoplasmatic environment (outside host cytoplasm) with feeder organelle at the base sitting on the apical surface of the small intestine [4]. While *C. hominis* infects only humans, *C. parvum* has multiple hosts and thus a zoonotic potential [5]. Given the infection route, the major strategy currently used to tackle cryptosporidiosis is prophylaxis by water treatment [6]. This is, however, hampered by the fact that oocysts are resistant to chlorine [7].

In immunocompetent individuals, *Cryptosporidium* infection is self-limiting and does not require a specific treatment, except prevention of dehydration. Currently, the only drug available against cryptosporidiosis is nitazoxanide, with limited efficacy [8] and potential side effects [9]. Consequently, hope is pinned on the development of novel chemotherapies, either via high-throughput screening of various compound libraries [10,11,12,13], or by de novo target-based drug development. Calcium-dependent protein kinases (CDPKs) with homologs in plants, apicomplexans and other phyla, but not in animals, are paradigmatic for such suitable targets. Investigations of CDPKs, in particular CDPK1 in *Toxoplasma gondii* [14] and other related apicomplexans [15], have resulted in the development of bumped kinase inhibitors (BKIs) of CDPK1 [16,17].

BKIs of *C. parvum* CDPK1 block infection of host cells in vitro [18] and cure cryptosporidiosis in vivo [19]. These findings suggest that these BKIs may be suitable anticryptosporidial drugs [20]. Other investigations showed, however, that there is a poor correlation between functional inhibition of the kinase and growth of *C. parvum* suggesting that CpCDPK1 is not essential at least not in vitro [21]. In vivo, the situation might be different [22]. More recent results showing that CpCDPK1 is only one of several CpCDPKs expressed in sporozoites [23], and the poor correlation between in silico docking studies, enzyme and proliferation inhibition [24], corroborate these results.

In vitro studies using *N. caninum* and *T. gondii* infected fibroblasts showed that BKIs induced the formation of multinucleated complexes composed of newly formed zoites lacking the outer plasma membrane, and therefore not able to separate and to form infective tachyzoites [25,26,27]. These findings suggest that BKIs could act on other targets besides CDPK1. Consequently, other mechanisms of action—perhaps host cell related—must be involved. In a previous study, we have shown that the 5-aminopyrazole-4-carboxamide (AC) BKI-1517 had a good efficacy against neosporosis in adult mice and interfered with vertical transmission but had detrimental effects on fertility in mice [28]. Similar results were obtained with the AC compound BKI-1748 affecting vertical transmission at lower dosage but interfering with fertility at higher dosage [27]. Moreover, several BKIs, including BKI-1748, caused embryonic malformations in a zebrafish (*Danio rerio*) model [29]. These BKIs share a quinoline core structure [29,30], which may at least be partially responsible for the side effects of these compounds since quinine, one of the first antiprotozoal drugs with this core structure, has long been known to have adverse side effects [31].

In the present article, we investigated whether (i) the treatment with BKI-1748 affects proliferation of *C. parvum* in vitro; (ii) the treatment of *C. parvum* infected host cells induces multinucleated complexes as observed with *N. caninum* and *T. gondii*; and (iii) BKI-1748 interacts with common potential targets in *C. parvum* and in host cells, and whether the quinoline core structure plays a role in this interaction. For this, we performed in vitro treatment studies followed by ultrastructural investigations of treated and control cultures and differential affinity chromatography followed by mass spectrometry with BKI-1748 and quinine on cell-free extracts from infected and uninfected human colon tumor cell line HCT-8 host cells. Inducing excystation and infecting 3 h prior to addition of compounds improves synchronization in the infected host monolayer. Subsequent incubation during 45 h permits investigation the effect of the compounds at key stages of the parasite life cycle such as invasion, egress/reinvasion, DNA replication, and sexual differentiation [32]. Moreover, gene expression spikes within this time frame [33].

## 2. Results

### 2.1. Inhibition of C. parvum Proliferation In Vitro by BKI-1748

**Figure 1:**
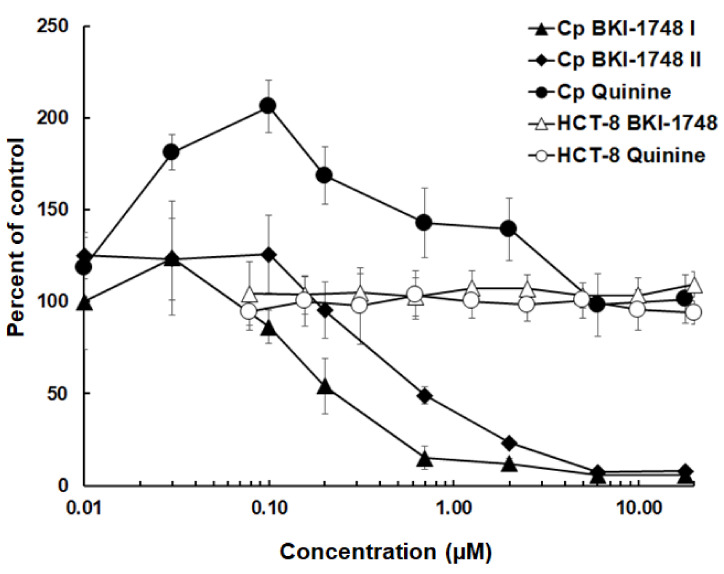
Dose-dependent effects of BKI-1748 and quinine on proliferation of *C. parvum* (Cp) and on uninfected host cells (HCT-8). Parasite loads and host cell viability were determined as described in Materials and Methods and are presented as the percentage of the corresponding solvent controls. Mean values ± SD are given for quadruplicates. The effects of BKI-1748 were assayed twice on infected cells (Cp BKI-1748 I and II).

In HCT-8 cell cultures infected with *C. parvum*, parasite proliferation was inhibited by BKI-1748 at sub-micromolar concentrations. Quinine sharing the same quinoline core structure had no inhibitory effects within the time frame and concentration range tested. Host cells were not affected by either compound in the concentration range tested (Figure 1).

The dose response for BKI-1748 was assayed twice, yielding an IC_50_ of 0.14 µM with a 95% error margin of 0.08–0.26 µM in the first assay and an IC_50_ of 0.24 µM with a 95% error margin of 0.14–0.4 µM in the second assay. Given the complex methodology of parasite quantification, both assays were in good agreement, and we considered 0.25 µM as valid estimation for the IC_50_ of BKI-1748 against *C. parvum* in HCT-8 cell cultures.

### 2.2. Scanning and Transmission Electron Microscopy of C. parvum Infected HCT-8 Host Cells Treated or Not with BKI-1748

**Figure 2:**
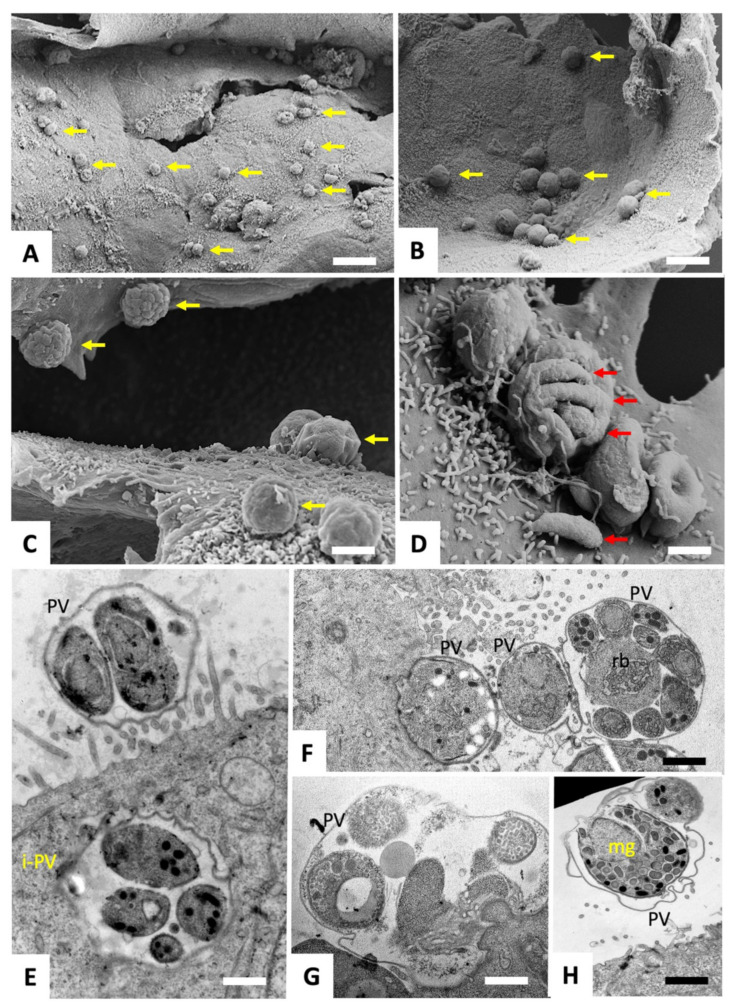
SEM (**A**–**D**) and TEM (**E**–**H**) of *C. parvum* grown in HCT-8 cells, fixed and processed at 48 h post infection. The parasitophorous vacuoles are easily discernible on the surface of HCT-8 cells by SEM ((**A**–**C**), yellow arrows), and individual merozoites can be seen at higher magnification ((**D**), red arrows). In (**E**), TEM demonstrates the presence of a surface-exposed parasitophorous vacuole (PV) and an intra-cytoplasmatic vacuole containing numerous merozoites (iPV). (**F**) shows a tangential cut through three neighboring schizonts, with the one on the far right exposing eight merozoites connected to the residual body (rb). (**H**) depicts a vacuole harboring a macrogamete. Bars in (**A**) = 10 µm; (**B**) = 5 µm; (**C**) = 1.8 µm; (**D**) = 1 µm; (**E**) = 0.6 µm; (**F**) = 0.8 µm; (**G**) = 0.5 µm; (**H**) = 1 µm.

Three hours after infection of HCT-8 monolayers with freshly excysted *C. parvum* sporozoites, cultures were treated with either BKI-1748 (2.5 µM, i.e., ten times the estimated IC_50_) or the corresponding amount of DMSO for 48 h. In parallel, BKI-1748 or DMSO were added to uninfected HCT-8 cells. Subsequently, samples were processed for SEM and TEM to investigate potential drug-induced alterations. SEM and TEM of infected and DMSO-treated HCT-8 monolayers fixed and processed at 48 h post infection (p.i.) are shown in Figure 2.

SEM demonstrated that parasites had formed numerous and easily discernible surface exposed parasitophorous vacuoles (Figure 2A–D). In some instances, the outer surface of the parasitophorous vacuole was distorted, allowing a glimpse into the vacuole and of individual merozoites, of which one was adhering onto the host cell surface (Figure 2D). TEM confirmed the presence of merozoites within these surface exposed vacuoles (Figure 2E–H), but also provided evidence that parasites, in few instances, also formed non-surface-associated vacuoles that were localized within the cytoplasm (Figure 2E).

**Figure 3:**
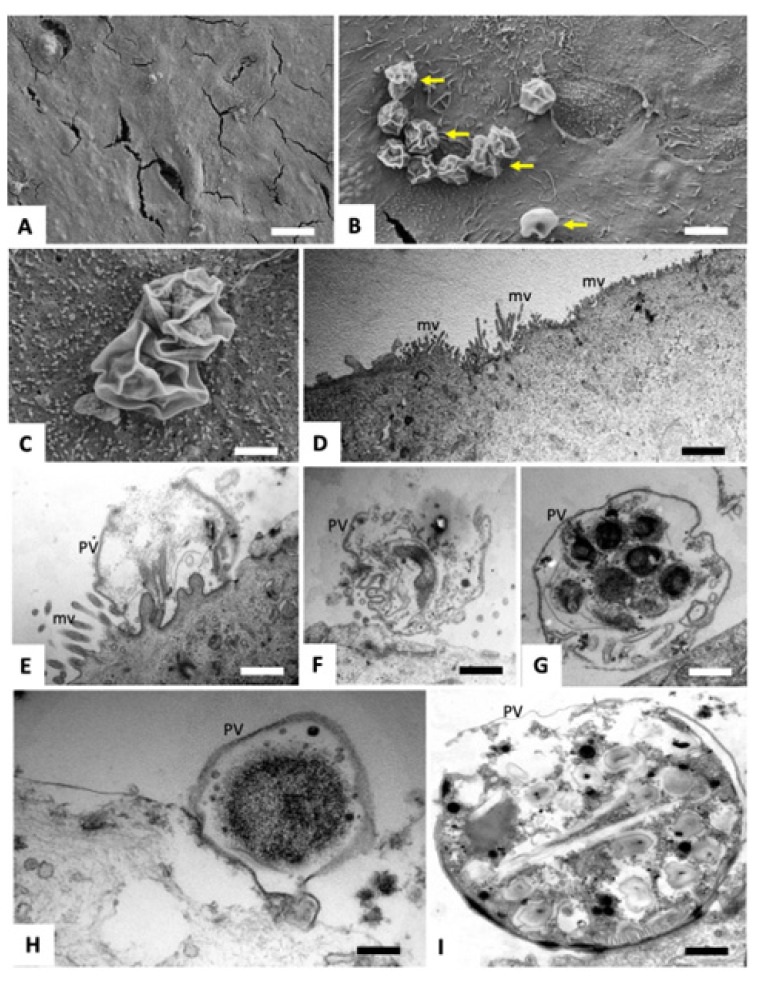
SEM (**A**–**D**) and TEM (**E**–**I**) of *C. parvum*-infected HCT-8 cells treated with BKI-1748 starting at 3 h post infection, and fixed and processed 45 h later. (**A**–**D**) shows that the surface of HCT-8 cells was largely devoid of parasitophorous vacuoles (PV), and in some instances collapsed vacuolar membranes could be seen (yellow arrows). Where visible, TEM demonstrates completely distorted PVs (**E**,**F**), few containing either distorted parasites (**G**) or just an amorphous mass (**H**). (**I**) could show a vacuole containing a potential gamete stage, although structurally heavily impaired. Bars in (**A**) 10 µm; (**B**) 2.8 µm; (**C**) 1 µm; (**D**) 2 µm; (**E**–**G**) 0.8 µm; (**H**,**I**) 0.5 µm.

Infected cells treated with BKI-1748 lost most parasitophorous vacuoles, and those remaining were exhibiting a collapsed structure as evidenced by SEM, with only membrane remnants present (Figure 3A–C).

TEM showed that the microvillar structures of the host cells were still in place (Figure 3D). Impairment of the host cells could not be noted, while the structural organization of the parasites within the few parasitophorous vacuoles that could be visualized was dramatically distorted. No multinucleated complexes were formed, instead the treatment with BKI-1748 resulted in rapid death and degradation, producing distorted parasite remnants. No viable parasites could be detected (Figure 3E–I).

### 2.3. DAC Proteomes of Uninfected and C. parvum Infected HCT-8 Host Cells

#### 2.3.1. Overview

**Figure 4:**
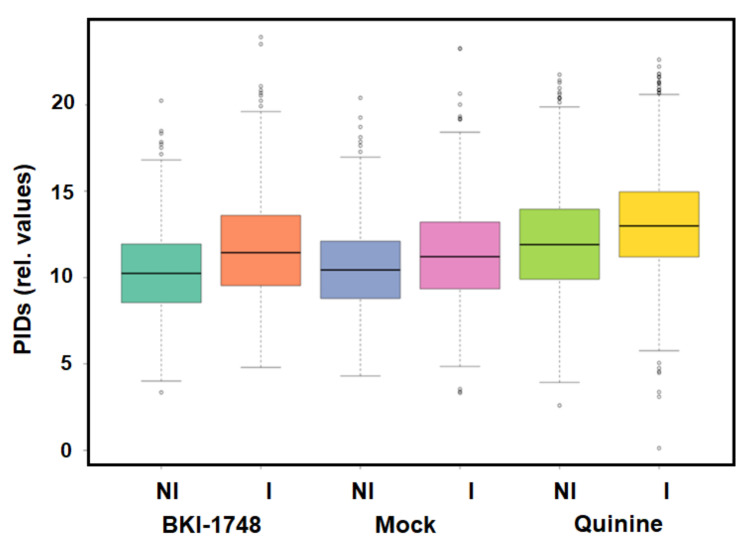
Protein intensity distributions (PIDs) of the proteome dataset presented by Table S1 as calculated by the iBAQ algorithm. Cell-free extracts of non-infected (NI) and infected (I) HCT-8 cells were prepared and subjected to differential affinity chromatography on mock, quinine, or BKI-1748 columns followed by mass spectrometry as described in Section 4.

Mass spectrometry analysis of the proteomes obtained after DAC of cell-free extracts of infected and uninfected HCT-8 host cells resulted in the identification of 29,684 unique peptides matching to 2725 host cell proteins and of 1389 unique peptides matching to 162 *C. parvum* proteins. The complete dataset is given in Table S1, which is available online. The protein intensity distributions were in the same range for all column eluates (Figure 4).

#### 2.3.2. *C. parvum* Proteins Identified by DAC

**Figure 5:**
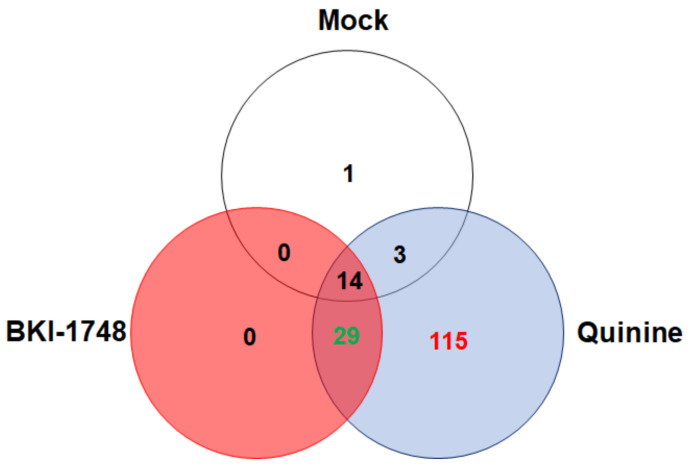
Venn diagram detailing the number of *C. parvum* proteins identified by DAC in cell-free extracts of infected HCT-8 host cells. Eluates from BKI-1748 and quinine columns were compared by MS shotgun analysis as described in Section 4. The numbers of the proteins identified within the subsets are explained in detail in the text.

A more detailed analysis of the 162 *C. parvum* proteins revealed that the by far biggest proportion, namely 115 proteins were identified in eluates from quinine columns only. A much smaller number, namely 29 proteins, were shared between BKI-1748 and quinine column eluates. In mock column eluates, only 18 proteins were identified, most of them shared with other column eluates. Interestingly, no binding proteins specific for BKI-1748 were identified (Figure 5). A complete list of the *C. parvum* proteins identified in this study is given in Table S2.

Within the subset of 115 binding proteins found in quinine column eluates only, the most abundant protein was a cold-shock DNA-binding domain protein followed by thioredoxin and a 60S ribosomal protein. Amongst the twenty most abundant binding proteins, eight were homologs to ribosomal proteins. Five other proteins shared homologies with DNA or nucleotide-binding proteins (Table 1).

Furthermore, 29 *C. parvum* proteins were identified in eluates from both quinine and BKI-1748, but not from mock columns. There were no proteins exclusively found in BKI-1748 eluates. Amongst these 29 proteins were 16 ribosomal proteins (Table S2). Eight proteins were more abundant in BKI-1748 than in quinine column eluates. The 60S ribosomal protein L39 is—by far—the most abundant of these proteins, as listed in Table 2.

The binding protein with the highest ratio of abundance between the BKI-1748 and quinine-binding protein subsets was an uncharacterized protein sharing homologies with a chaperone-like ATPase [34] involved in replication [35].

#### 2.3.3. Host Cell Proteins Identified by DAC

**Figure 6:**
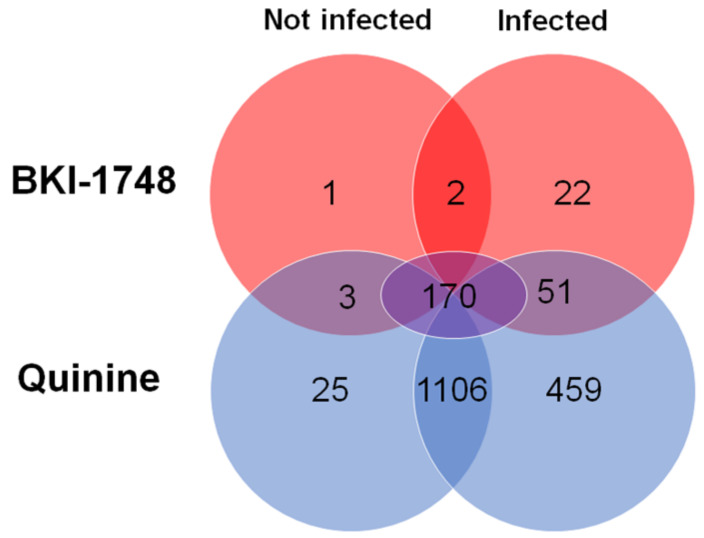
Venn diagram detailing the number of HCT-8 proteins identified by DAC in cell-free extracts of not infected and infected cells. Eluates from BKI-1748 and quinine columns were compared by MS shotgun analysis as described in Materials and Methods. The numbers of the proteins within the subsets are explained in detail in the text.

As mentioned above, 2725 host cell proteins were identified—1910 of them were not found in mock column eluates. The largest subset of these proteins, 1106 proteins, was identified in quinine column eluates from both non-infected and infected cells. A total of 459 proteins were identified in quinine column eluates from infected cells only. The third largest subset, namely 170 proteins, corresponded to proteins identified in BKI-1748 and quinine column eluates from both not infected and infected cells. Contrary to the *C. parvum* proteins, 25 binding proteins specific for BKI-1748 could be identified—1 in uninfected cells only, 2 in both uninfected and infected cells, and 22 in infected cells only (Figure 6). A complete list of the host cell proteins identified in this study is given in Table S3.

When looking at the specific BKI-1748-binding proteins in detail, it is striking that the pattern of not infected and infected cells was completely different. The most abundant BKI-1748 specific (and unique) protein from non-infected cells was the DNA repair protein XRCC1, whereas the protein S100-A2, a member of a family of calcium-binding proteins [36], was the most abundant BKI-1748-specific binding protein in infected cells. Two proteins were common to eluates from infected and non-infected cells (Table 3). One is Ladinin-1, a basement membrane protein probably involved in lung cancer formation [37]. The other is the isoform 2 of a specifically androgen-regulated protein, a steroid responsive transcription factor involved in prostate cancer and thyroid cancer metastasis [38].

When looking at proteins found in eluates from both quinine and BKI-1748 columns in infected and not infected cells, the numbers are much higher, namely 3 in not infected cells and 51 in infected cells only, as well as 170 in both non-infected and infected cells (Figure 6). Of the 170 common proteins, only 6 had higher abundances in BKI-1748-column eluates from both non-infected and infected cells than in the corresponding quinine column eluates. The Map-kinase-regulated corepressor-interacting protein 1 was the most abundant of these six proteins, followed by isoform 2 of the splicing factor 1, the protein with the highest abundancy ratio between the “BKI-1748 infected” and “quinine infected” binding protein subsets (Table 4).

The most abundant protein identified in host cell eluates from both BKI-1748 and quinine columns was the protein transport protein Sec23B, followed by the ribonucleoprotein A3 and the transport protein Sec23A. Moreover, five ribosomal proteins and four other proteins involved in RNA processing were amongst these twenty proteins (Table 5). 

Overall, when evaluating the potential functions of host cell and *C. parvum* proteins binding to both BKI-1748 and quinine, it was evident that most drug-binding proteins were related to RNA-binding and modification, including ribosomal proteins and proteins involved in RNA splicing, namely 72 of 224 host cell proteins and 16 (all ribosomal proteins) of 29 *C. parvum* proteins (Table 6).

## 3. Discussion

The quinoline core bumped kinase inhibitor BKI-1748 inhibits *C. parvum* proliferation in HCT-8 host cells with an IC_50_ of approximately 0.25 µM, thus the sub-micromolar range. Quinine sharing the same quinoline core structure neither affects *C. parvum* nor host cells in the concentration and time frames tested. This IC_50_ is one order of magnitude lower than the IC_50_ of nitazoxanide [39,40] and more than three orders of magnitude lower than the IC_50_ of paromomycin [39,41]. The coccidiostat lasalocid inhibits proliferation in the same order of magnitude as BKI-1748 [41]. In a more recent in vitro study, the miltefosine analog oleylphosphocholine inhibits *C. parvum* with an IC_50_ of approximately 20 nM, thus one order of magnitude lower than BKI-1748 [42].

Addition of BKI-1748 post infection is corelated with a dramatic inhibition of parasitophorous vacuole formation and irreversible damage of the ultrastructure of the merozoites as evidenced by TEM. This differs from the effects on intracellular *N. caninum* and *T. gondii* tachyzoites, which differentiate into multinucleated complexes, maintain viable for extended time periods and will back-differentiate into tachyzoites upon release of the drug pressure. Thus, interference with other targets than CDPKs may be responsible for the rapid effects of this compound on *Cryptosporidium*.

When looking at our differential chromatography results, it becomes striking that no *C. parvum* proteins and only twenty-five host cell proteins are exclusively identified in the BKI-1748 column eluates. Two of them, Ladinin-1 and the SARG protein, both related to cancer formation [37,38], are commonly found in non-infected and infected host cell eluates, while twenty-two proteins are identified in infected HCT-8 eluates only. The by far most abundant of them is the protein S100-A2. Identified in a screening for tumor suppressor genes and present in low levels in normal cells [36], S100-A2 is stimulated in keratinocytes by epidermal growth factor [43]. Upon binding of two Ca^2+^ ions, S100-A2 interacts with various proteins including tropomyosin and the tumor suppressor protein TP53. S100-A2 is downregulated in some tumors and upregulated in others (including colon carcinoma), thus high protein expression levels cannot be directly correlated to tumor formation [44]. It is unclear whether S100-A2 is upregulated in *C. parvum* infected cells what would explain its identification in infected cell eluates only. In a comprehensive study on differential expression of HCT-8 genes upon *C. parvum* oocyst infection, the S100-A2 transcript has not been listed amongst the differentials [45].

Nevertheless, it is unlikely that binding to these host cell proteins is responsible for the rapid action of BKI-1748 on *C. parvum*. The subsets of *C. parvum* proteins binding to both quinine and BKI-1748 provide better candidates to explain the rapid and devastating effect this drug exerted on *Cryptosporidium*. Among these, ribosomal proteins constitute the most dominant fraction, with 60S ribosomal protein L39 as the predominant BKI-1748 binding protein. According to text-book knowledge, the eukaryotic ribosome consist of four ribosomal RNAs and approximately 80 ribosomal proteins organized in the two subunits 60S and 40S [46]. The 60S subunit catalyzes the peptide bond formation and is therefore the target of translation inhibitors such as cycloheximide [47]. Since interference with translation is one of the usual suspects of a rapid mode of action of anti-infective compounds, we may safely postulate that BKI-1748 does exactly this in *C. parvum* and—to different extents—in other cells. Confirming our initial hypothesis that common targets may exist in *C. parvum* and in host cells, RNA binding and modifying proteins constitute predominant subsets of proteins binding to both quinine and BKI-1748 in HCT-8 host cells, as well. In addition to ribosomal proteins, this subset contains essential components of the spliceosome such as small ribonucleoproteins and pre-mRNA splicing factors [48]. Homologs of these proteins have been identified in a previous publication as BKI-1748 interaction partners in *Neospora caninum* and *Danio rerio* [30]. These results emphasize the role BKI-1748 and other compounds with quinoline cores could have on RNA splicing and translation. Interference with these pathways may explain short-term effects on *Cryptosporidium* and long-term effects on hosts.

Why does BKI-1748, but not quinine, exert such rapid effects on *C. parvum*? The rea-son may be an increased uptake of the first or a more efficient metabolism of the second compound. Moreover, this difference may be due to better interactions with some of the binding proteins. Candidates are the ribosomal protein L39 or the AAA+-ATPase homolog, the protein with the highest BKI-1748 vs. quinine-binding ratio. The role of this protein in *C. parvum* is unknown. In human cells, this protein forms a complex with the DNA polymerase eta and is essential for replication via this polymerase, most likely by dissolving DNA-protein complexes [35]. Moreover, specific interaction of BKI-1748 with host cell components may exert indirect effects on the parasite.

Taken together, our study underlines—once again—that target-based drug development is not a guarantee for safe drugs without side effects and that complex interactions with target organism and host cell components should be evaluated. Keeping in mind that the binding of a drug to a given protein does not automatically imply functional inhibition, the identification of affino-proteomes not only to the compound of interest, but also to compounds sharing structural similarities has the potential to avoid adverse side effects by subsequent steps of optimization.

## 4. Materials and Methods

### 4.1. Tissue Culture Medium, Biochemicals and Compounds

Cell culture medium was purchased from Gibco-BRL (Zürich, Switzerland), and biochemical agents except BKI-1748 were procured from Sigma (St. Louis, MO, USA). BKI-1748 was synthesized at the University of Washington and shipped as powder. Quinine and BKI-stock solutions (20 mM) were prepared in DMSO and were stored −20 °C until used.

### 4.2. In Vitro Culture and Processing of Parasites

For drug efficacy tests and microscopy, HCT-8 cells were maintained in RPMI 1640 medium containing 10% fetal bovine serum at 37 °C and a 5% CO_2._ For infection, *C. parvum* oocysts were centrifuged at 14,000 rcf for 4.5 min. To trigger excystation of sporozoites from these oocysts, the pellets were resuspended in 300 µL 10 mM HCl and incubated at 37 °C for 10 min. This was followed by another centrifugation step (14,000 rcf for 4.5 min), the pellets were resuspended in 300 µL 2 mM sodium taurocholate and incubated for 10 min at 16 °C [49]. Then, the oocysts in taurocholate were added to 10 mL RPMI 1640. For scanning electron microscopy (SEM), 2.5 × 10^6^ oocysts suspended in 1 mL medium per well were added to HCT-8 cells grown on glass coverslips in 24-well plates. For transmission electron microscopy (TEM), 10^7^ oocysts suspended in 5 mL medium per well were added to HCT-8 cells grown in 6-well plates. For affinity chromatography, 5 × 10^8^ oocysts suspended in 20 mL medium were added to HCT-8 cells grown in T-75 flasks.

### 4.3. Drug Efficacy Tests

The efficacy of BKI-1748 and quinine against *C. parvum* was analyzed using high-content microscopy as previously described [32,50]. Briefly, HCT-8 cells were grown to ~90% confluency in 384-well plates, and 2.5 × 10^5^ oocysts prepared as described above were added in 0.1 mL medium per well. Sporozoites released from excysted oocysts were allowed to invade host monolayers for 3 h. Then, different concentrations of BKI-1748, quinine and DMSO as a solvent control were added to that infected host cell monolayers, and the plates were incubated for another 45 h.

To prepare samples for quantification via fluorescence microscopy, wells were washed thrice with PBS containing 111 mM D-galactose (PBS-D-gal) followed by fixation with 4% paraformaldehyde (PFA) in PBS for 15 min at room temperature. Cells were permeabilized utilizing 0.25% Triton X-100 for 10 min at 37 °C then washed thrice with PBS-0.1% Tween 20. The cells were blocked with 4% bovine serum albumin (BSA) in PBS. To stain parasitophorous vacuoles, 1.33 µg/mL of fluorescein-labeled *Vicia villosa* lectin (Vector Laboratories, Newark, CA, USA; catalog# FL-1231) was diluted in 1% BSA:PBS:0.1% Tween 20 and added to each well followed by a 1 h at 37 °C. To stain for host cells, Hoechst 33258 (AnaSpec, Fremont, CA, USA; catalog# AS-83219) diluted at 0.09 mM diluted in water was added to each well followed by incubation for another 15 min at 37 °C. Prior to imaging, wells were washed 5 times with PBS containing 0.1% Tween 20.

A Nikon Eclipse Ti2000 epifluorescence microscope programmed using NIS-Elements Advanced Research software (https://www.microscope.healthcare.nikon.com/products/software/nis-elements/nis-elements-advanced-research; 1 January 2024; Nikon, Toko, Japan) was used take a 3 × 3 composite image using an EXi blue fluorescence microscopy camera (QImaging, Surrey, BC, Canada) and a 20 × objective (NA = 0.45). Host cell nuclei and parasite images were exported separately, and as .tif files. Analysis was performed utilizing macros developed on the ImageJ platform, version 1.54h (National Institutes of Health; Bethesda, MD, USA). Parasite numbers were normalized to DMSO control (% inhibition) and plotted on GraphPad Prism version 10.1.1. IC_50_ values were derived from non-linear regression analysis to determine dose-response.

Drug tests on not-infected host cells were performed by growing HCT-8 cells in 96-well plates to confluence. Then, concentration series of BKI-1748, quinine or DMSO as a solvent control were added, the plates were incubated for 72 h, and the cell vitality was determined using the resazurin reduction or AlamarBlue assay [51] as described [52]. Inhibition constants (IC_50_) were calculated by a logit-log algorithm as described [52].

### 4.4. Scanning and Transmission Electron Microscopy (EM) 

HCT-8 cells grown on glass coverslips for SEM or in 6 well plates for TEM were infected with excysted *C. parvum* oocysts as described above. After 3 h, BKI-1748 (2.5 µM) or the corresponding concentration of DMSO was added, and cultures underwent continuous treatment at 37 °C, 5% CO_2_ during 45 h. Specimens were fixed as previously described. For TEM, primary fixation was carried out in 100 mM sodium cacodylate pH 7.3/2% glutaraldehyde for 4 h at room temperature, and adherent cells were removed with a rubber cell scraper. Following centrifugation, post fixation was carried out in 2% osmium tetroxide in cacodylate buffer during 2 h. After several washes in distilled water, specimens were dehydrated in ethanol, and embedded in Epon 812 epoxy resin as previously described [53]. Following polymerization of the resin at 60 °C overnight, ultrathin (80 nm) sections were cut using an ultramicrotome (Reichert and Jung, Vienna, Austria). Sections were transferred onto formvar-carbon-coated 200 mesh nickel grids (Plano GmbH, Marburg, Germany), stained with Uranyless^®^ and lead citrate (both from Electron Microscopy Sciences, Hatfield, PA, USA), and imaging was performed on a FEI Morgagni TEM equipped with a Morada digital camera system (12 Megapixel) operating at 80 kV. For SEM, all fixation and dehydration steps were performed on glass coverslips. After a final dehydrations step in 100% ethanol, samples were twice immersed in hexamethyl-disilazene and were air-dried. Specimen were sputter coated with gold and inspected on a Zeiss Gemini450 SEM operating at 5 kV.

### 4.5. Protein Extraction and Differential Affinity Chromatography

HCT-8 cells infected with excysted oocysts as described above were harvested 48 h post infection by scraping, followed by centrifugation (500 rcf, 10 min, 4 °C), suspension of the pellets in PBS and subsequent centrifugation. In parallel, uninfected host cells were harvested, as well. Pellets were stored at −80 °C until further processing. Protein extraction and differential affinity chromatography was performed using mock, quinine and BKI-1748 columns as previously described [30]. Cell-free extracts of not infected and infected host cells were analyzed on separate sets of columns.

### 4.6. Proteomic Analysis of the Eluted Proteins by Mass Spectrometry

The lyophilized eluates were dissolved in 10 μL of 8 M urea and 0.1 M of Tris-HCl- (pH = 8), then, 1 μL of 0.1 M of Tris-Cl- (pH = 8) buffer containing 0.1 M of dithiothreitol were added, followed by incubation for 30 min at 37 °C and constant mixing with 600 rpm. This step was repeated with 1 μL of 0.5 M of iodoacetamide. Iodoacetamide was quenched by the addition of 5 μL 0.1 M of Tris-Cl- (pH 8), 0.1 M DTT, and the urea concentration further diluted to 4 M by the addition of 2 mM Calcium dichloride in 20 mM Tris buffer. Proteins were digested for 2 h at 37 °C by the addition of 1 μL of 0.1 μg/μL LysC sequencing grade protease (Promega, Madison, WI, USA), followed by further dilution of urea to 1.6 M with above Calcium dichloride buffer and 1 μL of 0.1 μg/μL trypsin sequencing grade (Promega). Digestion was completed by incubation over night at ambient room temperature. Digestion was stopped with 2.5 μL of 20% (*v*/*v*) trifluoroacetic acid. After an incubation for 15 min at room temperature, the digest was spun for 1 min at 16,000 g, and the cleared supernatant was transferred to a HPLC vial for subsequent nano-liquid reversed phase chromatography coupled to tandem mass spectrometry. system consisting of a Dionex Ultimate 3000 (ThermoFisher Scientific, Reinach, Switzerland) coupled to a timsTOF Pro through a CaptiveSpray source (Bruker, Bremen, Germany) with an endplate offset of 500 V, a drying temperature of 200 °C, and with the capillary voltage fixed at 1.6 kV. A volume of 2 µL from the protein digest was loaded onto a pre-column (PepMap 100 C18, 5 µm, 100 A, 300 µm diameter × 5 mm length, ThermoFisher) at a flow rate of 10 µL/min with 0.05% trifluoroacetic acid in water/acetonitrile 98:2. After loading, peptides were eluted in back flush mode onto a home-made C18 CSH Waters column (1.7 µm, 130 Å, 75 µm × 20 cm) by applying a 70-min gradient of 5% acetonitrile to 40% in water/0.1% formic acid, at a flow rate of 250 nL/min. The timsTOF Pro instrument was operated in data-dependent acquisition (DDA) mode using the Parallel Acquisition Serial Fragmentation (PASEF) option. The mass range was set between 100 and 1700 *m*/*z*, with 10 PASEF scans between 0.7 and 1.4 V s/cm^2^. The accumulation time was set to 2 ms, and the ramp time to 100 ms, respectively. Fragmentation was triggered at 20,000 arbitrary units, and peptides (up to charge of 5) were fragmented using collision induced dissociation with a spread between 20 and 59 eV.

The mass spectrometry data were processed with FragPipe software (v.20.0) [54] against concatenation of the following databases: Crypto protein sequence database downloaded from NCBI (June 2023), SwissProt Homo Sapiens (release 2023_04), and 230 proteins commonly found as contaminants; decoys were included as reversed sequences. Quantification was performed by the IonQuant algorithm; filtering of protein identification to a 1% false discovery rate (FDR) on the peptide level was performed by the Percolator algorithm.

Variable modifications of one oxidation on methionine, protein N-terminal acetylation, and deamidation on asparagine or glutamine were allowed together with fixed car-bamidomethylation of cysteine. Identified proteins were filtered by the criterium that at least two different razor peptide sequences were identified as evidence for the existence of the protein. iBAQ values were calculated in R in the following manner: the sum of peptide intensities was divided by the number of possible tryptic peptides that have 6 AA ≤ length ≥ 30 AA. Relative abundance (rAbu) was calculated based on the IBAQ values such that the sum of rAbu is equal to 10^6^ in each sample.

## Figures and Tables

**Table 1:** List of the twenty most abundant *C. parvum* proteins from infected HCT-8 host cells specifically binding to quinine columns, as identified by DAC followed by mass spectrometry. See Table S1 for the full dataset and Table S2 for the full subset of *C. parvum* proteins. The relative abundances (rAbu) based on iBAQ sum up to a total of 1,000,000 for each sample. The proteins are listed according to their decreasing rAbu values in quinine eluates.

Protein ID	Annotation	rAbu
QOY39990.1	Cold-shock DNA-binding domain-containing protein	2631
QOY40836.1	Thioredoxin	1687
QOY40770.1	60S ribosomal protein L6	1173
QOY42506.1	Profilin	983
QOY42741.1	60S ribosomal protein L30	565
QOY41364.1	Tubulin beta chain	562
QOY42473.1	Ribosomal protein L14	527
QOY40573.1	60S ribosomal protein L35A	463
QOY41580.1	Nucleoside diphosphate kinase	438
EAZ51383.1	Guanine nucleotide-binding protein, putative	404
QOY43535.1	Actin	395
QOY40892.1	Disulfide-isomerase, signal peptide plus ER retention motif	377
QOY43201.1	60S acidic ribosomal protein LP2	354
QOY41122.1	Divalent ion tolerance protein, CutA/nitrogen regulatory protein PII/ATP phosphoribosyltransferase	337
QOY41334.1	Uncharacterized protein with Armadillo-like helical	296
EAZ51402.1	60S ribosomal protein L13, putative	288
QOY40344.1	50S-L18Ae/60S-L20/60S-L18A ribosomal protein	286
QOY40195.1	Uncharacterized protein CPATCC_0004510	236
EAZ51528.1	Poly(a)-binding protein fabm, putative	216
QOY42201.1	60S ribosomal protein L31	208

**Table 2:** List of eight *C. parvum* proteins binding to both BKI-1748 and quinine columns with higher abundances in BKI-1748 column eluates, as identified by differential affinity chromatography followed by mass spectrometry. See Table S2 for the full list of *C. parvum* proteins. The relative abundances (rAbu) based on iBAQ sum up to a total of 1,000,000 for each sample. The proteins are listed according to their decreasing rAbu values in BKI-1748 eluates.

Protein ID	Annotation	rAbu BKI-1748	rAbu Quinine
QOY43223.1	60S ribosomal protein L39	1386	462
EAZ51504.1	Ribosomal protein S23, partial	51	43
QOY40793.1	Uncharacterized protein CPATCC_0010570 (RF-C Ctf18p AAA + ATPase)	48	3
QOY40752.1	Uncharacterized protein CPATCC_0010160	28	10
QOY41888.1	Hypothetical protein CPATCC_0017110	22	16
ABJ09792.1	Heat shock protein 70, partial	22	20
QOY40881.1	Uncharacterized protein CPATCC_0011490	21	13
QOY41279.1	Uncharacterized protein with tetratricopeptide-like helical (Garp protein)	9	1

**Table 3:** List of host cell proteins specifically binding to BKI-1748 columns, as identified by DAC followed by mass spectrometry. See Table S1 for the full dataset and Table S3 for the full subset of host cell proteins. The relative abundances (rAbu) based on iBAQ sum up to a total of 1,000,000 for each sample. The proteins are listed according to their decreasing rAbu values. *, proteins found in eluates from both not infected and infected HCT-8 cells.

Protein ID	Annotation	rAbu
	*Not infected cells*	
P18887	DNA repair protein XRCC1	100
Q9BW04-2	Isoform 2 of specifically androgen-regulated gene protein *	23
O00515	Ladinin-1 *	10
	*Infected cells*	
P29034	Protein S100-A2	259
P81605-2	Isoform 2 of dermcidin	84
P01036	Cystatin-S	83
P10909-2	Isoform 2 of clusterin	69
Q9UBH0	Interleukin-36 receptor antagonist protein	49
P56134-2	Isoform 2 of ATP synthase subunit f, mitochondrial	44
Q9H190-3	Isoform 3 of syntenin-2	40
Q01546	Keratin, type II cytoskeletal 2 oral	34
P01037	Cystatin-SN	33
P20933	N(4)-(beta-N-acetylglucosaminyl)-L-asparaginase	16
Q9UQB8-2	Isoform 2 of brain-specific angiogenesis inhibitor 1-associated protein 2	13
Q3ZCW2	Galectin-related protein	13
O14556	Glyceraldehyde-3-phosphate dehydrogenase, testis specific	12
P35580-2	Isoform 2 of myosin-10	12
Q96L46	Calpain small subunit 2	11
O95817	BAG family molecular chaperone regulator 3	10
Q9BY77-2	Isoform 2 of polymerase delta-interacting protein 3	8
Q8N6H7-2	Isoform 2 of ADP-ribosylation factor GTPase-activating protein 2	7
Q14157-1	Isoform 2 of ubiquitin-associated protein 2-like	6
O00515	Ladinin-1 *	4
Q93009-3	Isoform 3 of ubiquitin carboxyl-terminal hydrolase 7	4
P0DOX6	Immunoglobulin mu heavy chain	3
Q9BW04-2	Isoform 2 of specifically androgen-regulated gene protein *	3
O75592-2	Isoform 2 of E3 ubiquitin-protein ligase MYCBP2	1

**Table 4:** List of six host cell proteins binding to both BKI-1748 and quinine columns with higher abundances in BKI-1748 column eluates, as identified by differential affinity chromatography followed by mass spectrometry. See Table S3 for the full list of host cell proteins. The relative abundances (rAbu) based on iBAQ sum up to a total of 1,000,000 for each sample. The proteins are listed according to their decreasing rAbu values in BKI-1748 eluates. NI, not infected cells; I, infected cells.

Protein ID	Annotation	rAbu NI	BKI-1748 I	rAbu NI	Quinine I
C9JLW8	Mapk-regulated corepressor-interacting protein 1	221	38	47	26.5
Q15637-2	Isoform 2 of splicing factor 1	86	139	33	17.9
P29144	Tripeptidyl-peptidase 2	83	6	6	5.8
P36957	Dihydrolipoyllysine-residue succinyltransferase component of 2-oxoglutarate dehydrogenase complex, mitochondrial	22	8	5.9	6.2
Q13573	SNW domain-containing protein 1	17	13	17.0	7.9
Q9UHB6-4	Isoform 4 of LIM domain and actin-binding protein 1	16	11	8.5	5.6

**Table 5:** List of the twenty most abundant host cell proteins binding to both BKI-1748 and quinine columns all columns confounded, as identified by DAC followed by mass spectrometry. See Table S3 for the full list of host cell proteins. The relative abundances (rAbu) based on iBAQ sum up to a total of 1,000,000 for each sample. The proteins are listed according to their decreasing rAbu values in “quinine infected cells”. NI, not infected cells; I, infected cells.

Protein ID	Annotation	rAbu NI	BKI-1748 I	rAbu NI	Quinine I
Q15437	Protein transport protein Sec23B	94	19	1234	2780
P51991	Heterogeneous nuclear ribonucleoprotein A3	145	107	1212	1938
Q15436	Protein transport protein Sec23A	17	4	719	1735
Q13162	Peroxiredoxin-4	22	7	619	1036
Q86X55-1	Isoform 1 of histone-arginine methyltransferase CARM1	26	16	594	1016
Q14103-3	Isoform 3 of heterogeneous nuclear ribonucleoprotein D0	122	26	512	963
Q12906-7	Isoform 7 of interleukin enhancer-binding factor 3	28	23	731	928
O96019	Actin-like protein 6A	156	4	1435	883
Q15434	RNA-binding motif, single-stranded-interacting protein 2	38	37	1091	869
Q9Y2R9	28S ribosomal protein S7, mitochondrial	113	17	1660	836
Q9BUJ2-2	Isoform 2 of heterogeneous nuclear ribonucleoprotein U-like protein 1	129	217	401	782
A1KXE4	Myelin-associated neurite-outgrowth inhibitor	584	306	381	490
P61247	40S ribosomal protein S3a	390	206	790	384
P09496-2	Isoform non-brain of clathrin light chain A	139	36	630	375
P46779	60S ribosomal protein L28	672	264	819	337
P82930	28S ribosomal protein S34, mitochondrial	26	31	420	314
Q99460	26S proteasome non-ATPase regulatory subunit 1	16	370	326	165
P15880	40S ribosomal protein S2	81	10	291	146
Q3MHD2-2	Isoform 2 of protein LSM12 (NAADP receptor)	362	51	225	78
Q8WWM7	Ataxin-2-like protein	142	102	228	54

**Table 6:** Summary of putative functions of proteins binding both to quinine and BKI-1748 in *C. parvum* infected HCT-8 host cells. (see Tables S2 and S3 for complete lists). The functions were identified based on information given by Uniprot (www.uniprot.org) and related databases.

Function	HCT-8 Cells	*C. parvum*
DNA binding and modification	18	2
RNA binding and modification	72	16
Protein binding and modification	29	1
Cytoskeleton and intracellular transport	43	2
Intracellular signaling	18	0
Energy and intermediary metabolism	44	0
Hypothetical or ambiguous	0	8
Total	224	29

## Data Availability

Data are made available as Supplementary Materials (see above).

## Supplementary Materials

The following are available online at https://www.mdpi.com/article/10.3390/ijms25052707/s1.
